# P02.168. Safety issues and orthorexia in paediatrics

**DOI:** 10.1186/1472-6882-12-S1-P224

**Published:** 2012-06-12

**Authors:** I von Rosenstiel, J Stam, W Schats

**Affiliations:** 1 Slotervaart Hospital, Amsterdam, Netherlands

## Purpose

As an antithesis to the paediatric obesity pandemic, the eating disorder of orthorexia in parents and children is an emerging condition. Well-intentioned parents strictly limit their childrens’ food groups, be it carbohydrates, transfats, animal products, dyes or sugars, leading to dangerous extremes and malnutrition.

## Methods

We descibe our clinical experiences with families with extreme diets at our outpatient integrative medicine clinic in Amsterdam, the Netherlands. Data were analysed on parent and child demographics and clinical characteristics, family health concerns, educational level, lifestyle and social consequences.

## Results

During 12 months, a total of 4 cases of extreme diets were reported from a cohort of 41 patients. Two cases concerned sugar-free diets, one case of a vegan diet and one case of 100% raw food. Negative medical outcomes were nutritional deficiencies, stunting, concentration and developmental disorders, and social isolation. The raw food case involving a mother with orthorexia and her 14 year old son has led to a court case, causing a national debate on autonomy of the family versus child abuse.

## Conclusion

Pediatricians should be more knowledgable about the phenomenon of orthorexia and the potential warning signs. The so-called "worry factor" and "feelings towards food groups" are the biggest indicators to aid in preventive efforts. More studies in the pediatric field adressing effectiveness and safety issues of specific diets are needed to assist the experts in daily practice and medical court cases.

